# State or trait: the neurobiology of anorexia nervosa — contributions of a functional magnetic resonance imaging study

**DOI:** 10.1186/s40337-022-00598-7

**Published:** 2022-05-31

**Authors:** Selma Göller, Kathrin Nickel, Isabelle Horster, Dominique Endres, Almut Zeeck, Katharina Domschke, Claas Lahmann, Ludger Tebartz van Elst, Simon Maier, Andreas A. B. Joos

**Affiliations:** 1 Department of Psychosomatic Medicine and Psychotherapy, Medical Center - University of Freiburg, Faculty of Medicine, University of Freiburg, Freiburg, Germany; 2 Department of Psychiatry and Psychotherapy, Medical Center - University of Freiburg, Faculty of Medicine, University of Freiburg, Freiburg, Germany; 3 Department of Psychosomatic Medicine and Psychotherapy, Ortenau Klinikum, Lahr, Academic Teaching Hospital of the University of Freiburg, Lahr, Germany; 4 Center for Basics in Neuromodulation, Faculty of Medicine, University of Freiburg, Freiburg, Germany

**Keywords:** Anorexia nervosa, Recovery, State, Trait, Functional magnetic resonance imaging

## Abstract

**Background:**

The understanding of the cerebral neurobiology of anorexia nervosa (AN) with respect to state- versus trait-related abnormalities is limited. There is evidence of restitution of structural brain alterations with clinical remission. However, with regard to functional brain abnormalities, this issue has not yet been clarified.

**Methods:**

We compared women with AN (n = 31), well-recovered female participants (REC) (n = 18) and non-patients (NP) (n = 27) cross-sectionally. Functional magnetic resonance imaging was performed to compare neural responses to food versus non-food images. Additionally, affective ratings were assessed.

**Results:**

Functional responses and affective ratings did not differ between REC and NP, even when applying lenient thresholds for the comparison of neural responses. Comparing REC and AN, the latter showed lower valence and higher arousal ratings for food stimuli, and neural responses differed with lenient thresholds in an occipital region.

**Conclusions:**

The data are in line with some previous findings and suggest restitution of cerebral function with clinical recovery. Furthermore, affective ratings did not differ from NP. These results need to be verified in intra-individual longitudinal studies.

**Supplementary Information:**

The online version contains supplementary material available at 10.1186/s40337-022-00598-7.

## Plain English summary

There is abundant evidence of structural and functional brain alterations during the acute stage of anorexia nervosa (AN), although affected brain areas differ based on various study methodologies. Meanwhile, investigations indicate that brain structure normalizes with weight and clinical restitution. The current cross-sectional investigation examines acutely ill AN patients, healthy controls, i.e. non-patients (NP) and well-recovered individuals (REC), with respect to brain function. Functional cerebral responses of participants exposed to food pictures were investigated. Neither in terms of function nor emotional experience of food stimuli, the REC differed from the NP group. This study points to brain function normalizing with clinical and weight restoration, which should be verified in intra-individual longitudinal studies.

**Supplementary Information:**

The online version contains supplementary material available at 10.1186/s40337-022-00598-7.

## Background

Anorexia nervosa (AN) is an eating disorder largely affecting young women with high morbidity, chronicity and mortality [1]. Apart from a restriction of energy intake leading to a significant weight loss, an intense fear of gaining weight and body image disturbance are key symptoms. The etiology is not well understood, though genetic disposition is one important factor, accompanied by psychosocial factors [2]. Maintaining factors include consequences of malnutrition, as well as consequences of isolation and depression [3]. Brain imaging studies point towards shrinkage of white (WM) and grey matter (GM) and complementary increase of the cerebrospinal spaces [4, 5]. Longitudinal investigations show restitution of GM and WM volumetric alterations following long-term weight restoration [4]. Apart from structural brain alterations, also the function of various domains has been reported to be affected in acute AN [6, 7], although the involved brain areas differed [8]. Due to significant loss of weight, cerebral aberrations might be associated with metabolic changes, i.e. starvation, and it is difficult to disentangle other factors associated with eating disorder psychopathology and/or predisposing factors [9].

Hence, the question remains which abnormalities are state phenomena, i.e. occur only during the acute phase of the disease, and which are trait-related. Persistent cerebral aberrations could be a predisposing phenomenon and might therefore represent endophenotypes [10], but they may also represent sequels, i.e. “scars” of the acute disease. As AN is a disorder with low prevalence [2], it is methodically difficult to study individuals who might develop AN longitudinally in order to shed light on the question of endophenotypic cerebral aberrations. Therefore, a first step to clarify questions about state and trait is to investigate individuals recovered from AN (REC).

With respect to the issue of state-trait in AN, previous studies examined psychological, cognitive and behavioral variables [11, 12] as well as metabolic, structural and functional correlates of the brain [13–15]. Overall, research on reversibility of psychological, cognitive and behavioral functioning in REC presents a heterogeneous picture [16–18]. In terms of structural cerebral alterations, neuroimaging studies provide strong evidence of remission with clinical recovery [5, 19, 20]. Findings of functional magnetic resonance imaging (fMRI) studies comparing REC and non-patients (NP) show divergent results. With respect to visual food cues, some authors reported no alteration in brain activation in REC (when compared to NP) [21–23], while Uher et al. [24] detected increased activation of prefrontal and anterior cingulate cortices (ACC) and a reduction of activity in parietal regions in a small group of REC. Further studies found hypoactivation of the insula [25] or increased caudate activation [26] in response to food pictures. With regard to other disorder specific paradigms (e.g. taste, body shape), both increased and decreased functional neural brain responses for REC (compared to NP) were found in various brain regions [13, 27–30], while two studies detected no altered brain activity in REC [31, 32]. However, a majority of fMRI studies using non-disorder specific stimuli (e.g., fear, intimacy, reward) reported no or only minimal functional aberrations in REC [33–36]. Longitudinal fMRI studies with non-disorder specific paradigms yielded conflicting results in delay discounting tasks [37, 38], a normalization in a working memory and set-shifting task [39, 40], and persisting changes in theory of mind and reward learning paradigms [41, 42]. For further details and an overview of previous studies, see Additional file 1.

This investigation focuses on the question of restitution vs. non-restitution of functional brain abnormalities using a cross-sectional design in order to address the topic state and trait of the neurobiology in AN. In this context, the comparison of NP and REC is of particular interest. It complements previous studies on disease-specific food stimuli in REC [21–26]. The paradigm has already been employed previously in a study of AN with some REC participants [24, 43] and a study focusing on NP versus AN [44]. Data from the current study comparing NP and AN has been reported previously, with a focus on replicability issues [45]: Group comparisons yielded higher blood oxygenation-level dependent (BOLD) responses of AN compared to NP in midcingulate, pre/postcentral and parietal areas when using a lenient initial threshold, and no significant group differences with a conservative initial threshold.

Based on results of preceding whole-brain analyses [23, 25], we expected no differences between REC and NP in neural response to food stimuli or behavioral/experiential response, i.e., affective ratings of stimuli [22, 24, 46]. Additionally, we performed exploratory analyses of affective ratings and insula activation, as earlier studies found a positive correlation between food pleasantness ratings and insula activation in the NP group [22, 25], but not in the AN [25] or REC groups [22].

## Methods

### Participants

AN and REC participants were recruited via the Department of Psychosomatic Medicine and Psychotherapy of the University Medical Center Freiburg. NP were recruited via local advertisements. The study was performed following written informed consent from the participants. The data was collected between March 2015 and October 2017.

Thirty-one AN, 18 REC and 27 NP were included in the final analysis. AN participants had to fulfill DSM-5 criteria. The following inclusion criteria were defined for the REC group: (1) Absence of eating disorder symptomatology for more than 12 months and an Eating Disorder Examination (EDE) [47] within one standard deviation of normal; (2) The Body Mass Index (BMI) was aimed at ≥ 20 kg/m^2^, which we achieved for most REC. The BMI of four participants was slightly below 20 kg/m^2^ (between 19.3 and 19.7 kg/m^2^) and of one participant 18.8 kg/m^2^. We decided to include these participants because they were clinically completely recovered and had always had a BMI in this range before the onset of the disease. Three AN patients were of the binge eating/purging type, all other AN and REC were of the restrictive type. Patients with AN were seen in the outpatient clinic for diagnostic reasons while nine were right at the beginning of inpatient treatment. Exclusion criteria had been reported previously [45].

The participants examined in the current study largely overlap with those of our previous investigations [5, 14, 19, 34, 35, 45].

### Procedure

The study was approved by the local Ethics Committee (EK 520/13). Participants were assessed by means of the SCID interview [48, 49], the EDE [47, 50] and the following self-report questionnaires: Beck Depression Inventory-II (BDI-II) [51], Eating Disorder Inventory-2 (EDI-2) [52], State-Trait Anxiety Inventory (STAI) [53], and a crystalline intelligence test (MWT-B) [54]. All participants were studied in the second half of the menstrual cycle or the corresponding phase with estrogen and progesterone when taking oral contraception. In the morning around 8 a.m., participants were provided with a standardized breakfast, the calories consumed were counted, and the feeling of satiety was rated on a Likert-scale from 0 (very hungry) to 9 (very satiated).

### Paradigm

Participants viewed via a mirror photographs of food and non-food items of similar structure [43–45] presented on a BOLDScreen monitor at the rear of the scanner bore. In a block design with five blocks per condition of 30 s each, 10 consecutive food or non-food pictures were presented alternately per block.

**Fig. 1:**
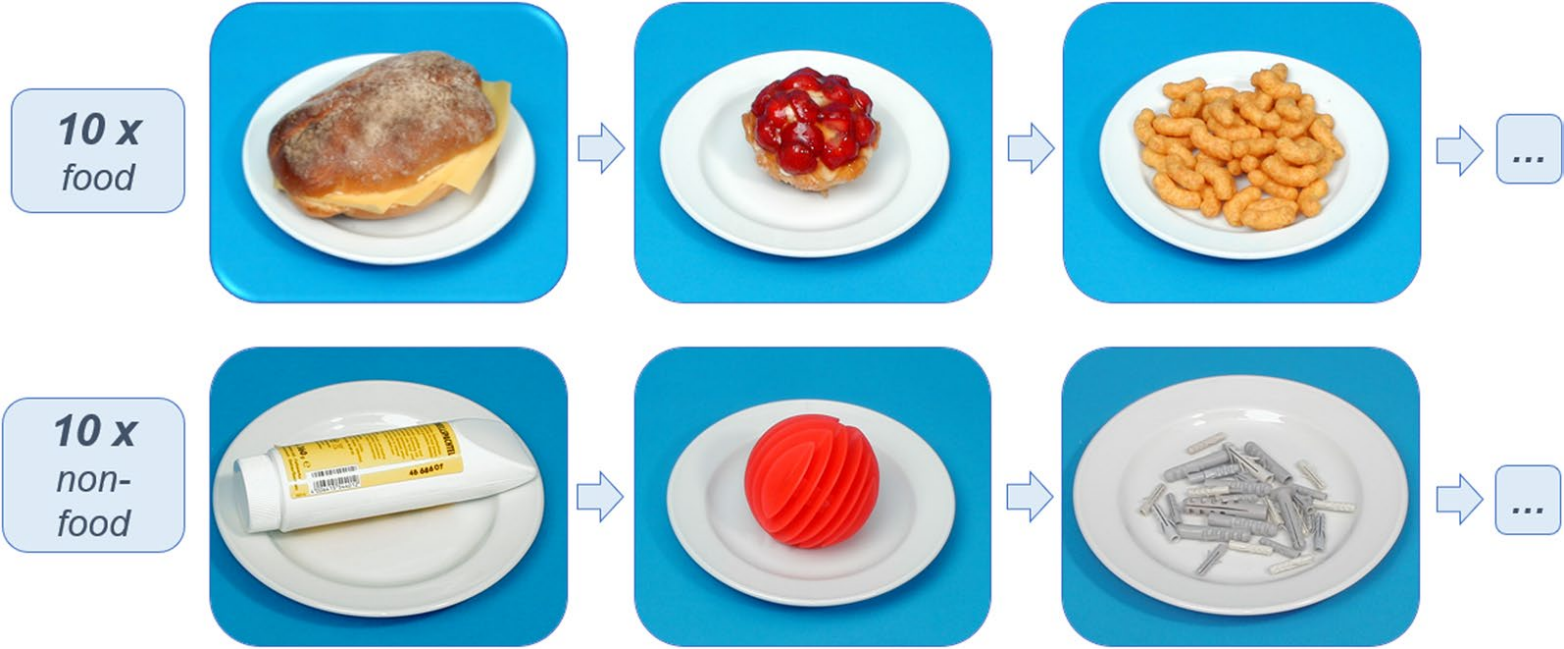
Examples of food and non-food stimuli (cf. [45])

Participants were asked to look attentively at the pictures. Examples of the picture stimuli are displayed in Fig. 1.

### Behavioral data

After scanning, participants rated a selection of images (10 food and 10 non-food images) with respect to three emotion dimensions (valence, arousal and dominance) using “manikin ratings” based on the International Affective Picture System on scales from zero to eight [54]. The dominance scale assesses how much the viewer feels controlled or in control when watching the images [55]. Ratings of two AN and two NP could not be included due to incomplete data.

### Image acquisition and processing

A T1-weighted Magnetization-prepared rapid gradient echo (MPRAGE) sequence (TR = 2300 ms, TE = 2.98 ms, flip angle = 9°, FOV = 240 * 256 mm^2^, voxel size = 1 × 1 × 1 mm^3^) was recorded as an anatomical reference. 138 functional echo-planar T2*-weighted (EPI) images (TR = 2500 ms, TE = 30 ms, flip angle = 90°, FOV = 192 * 192 mm^2^, Matrix volume = 64 × 64, 38 slices, voxel size = 3 × 3 × 3 mm^3^) were recorded with a Siemens 3 T Prisma MAGNETOM (Siemens Medical Systems, Erlangen) using a 20-channel head coil. All EPI images were automatically rigid-body transformed to correct for head motion and a distortion correction algorithm was applied [56]. Preprocessing and statistical analysis of the functional data was performed with the statistical parametric mapping software SPM12 (Wellcome Trust Centre of Imaging Neuroscience, London; for details, see [57]). The first two volumes of each run were disregarded and an artifact detection algorithm (ArtRepair toolbox, SPM) was applied to detect head motion and possible spiking artifacts. The functional raw images were realigned to the first volume to generate six head motion parameters (rotation and translation in x, y, z direction), which were used as regressors of no interest in the first-level statistical analysis to correct for influences of head motion. The ‘Artrepair’ tool implemented in SPM12 was used to correct movement artifacts over half a voxel size by interpolating the measurement time points before and after the movement. Participants whose head movements were larger than half a voxel size (corresponding to 1.5 mm for a voxel size of 3 × 3 × 3 mm^3^) were excluded from the analysis if more than two consecutive measurement time points (= "volumes") were affected or more than two corrections had to be made in the time series. In the case of spiking artifacts again the ‘Artrepair’ toolbox was used to correct single slices by interpolating the slice below and above the affected slice. If several slices of a single volume were affected, we interpolated (correspondingly to motion artifacts) the measurement time points before and after the volume affected by spiking artifacts. If two consecutive volumes or more than two volumes in total were affected by spiking artifacts, the subject was excluded. The motion corrected images were spatially normalized into the MNI (Montreal Neurological Institute) reference system applying the anatomical MPRAGE image. To increase the signal-to-noise ratio and to compensate for inter-individual differences in location of corresponding functional areas, the data was spatially smoothed with a three-dimensional isotropic Gaussian kernel (8 mm FWHM). Low frequency artifacts across the time-series were removed applying a high-pass filter (128 s).

### Statistical analysis

Demographic, clinical and behavioral data were assessed by analyses of variance with a level of significance of *p* < 0.05 (two-sided).

Food and non-food regressors were convolved with a canonical hemodynamic response function and fitted together with the six regressors for head motion parameters in a linear regression model (general linear model (GLM)) with the functional signal time courses for each voxel and participant.

### Within-group activation

In the second-level whole brain analysis, we tested for within-group differences (group activation) by performing a one-sample *t*-test for the food > non-food contrast of the first-level beta estimates of the food and non-food regressors.

### Between-group comparison

For group comparisons, the first-level food > non-food contrast was used to compare AN > REC, REC > AN, NP > REC, and REC > NP in a two-sample t-test.

For both, the within- and between-group analysis: (1) We added age as a covariate. (2) We performed whole brain analyses with a cluster-defining threshold, i.e., initial height threshold, of *p*_uncorr._ < 0.001 and a minimum cluster size of 10 voxels (k ≥ 10) (3) Results were corrected for multiple comparisons on a cluster level applying family-wise error correction with a threshold of *p*_corr._ < 0.05.

For the between-group analysis, we additionally performed analyses with a cluster-defining threshold of *p*_uncorr._ < 0.01 and a minimum cluster size of 10 voxels, as previous studies had used lenient thresholds of *p*_uncorr._ < 0.01 [24, 44] or even lower (*p*_uncorr._ < 0.05) [22, 25].

Moreover, we performed ROI-based (region of interest) small volume correction (SVC) for insula and amygdala ROIs according to the AAL3 atlas [58].

Comparisons of the AN and NP group are not reported as they have already previously been published [45].

### Multiple regression analysis

For all groups and for each group separately, SPM multiple regression analyses were performed to calculate the correlation of the first-level BOLD contrast (food and non-food) with the valence and arousal ratings of the food stimuli. We tested for positive and negative linear effects of stimulus ratings and included the factor group as a regressor of non-interest (to adjust for possible group differences). We set up an interaction model with the factors group and stimulus ratings as regressors of interest and age as regressor of non-interest and compared the regression slopes of AN > NP, AN > REC, and NP > REC and vice versa.

### Behavioral data

We set-up three separate Analysis of Variance (ANOVA) models for valence/arousal/dominance (dependent variable) with the independent factor group followed by post-hoc Tukey Kramer tests to assess for between-group differences.

## Results

### Participants

**Fig. 2:**
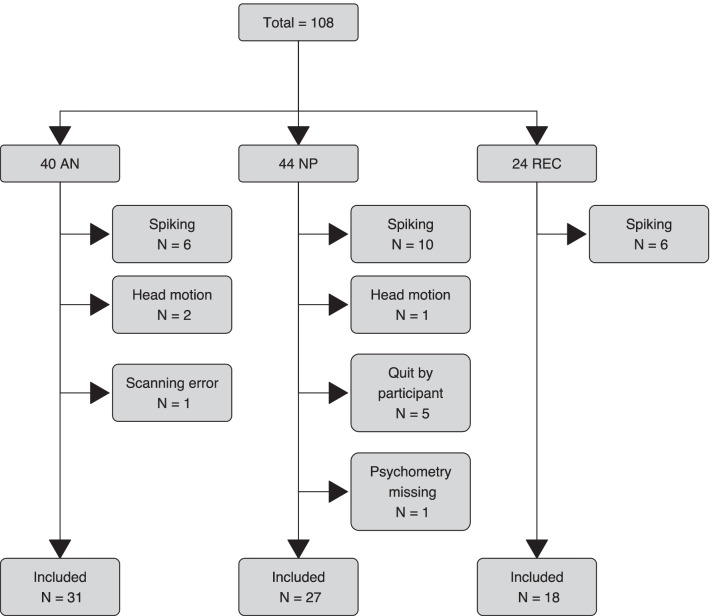
Exclusion flow chart. *AN* anorexia nervosa, *NP* non-patients, *REC *recovered AN

One hundred and eight female participants (40 AN, 24 REC, 44 NP) were recruited. Thirty-two data sets had to be discarded due to insufficient data quality, spiking head motion or incomplete data (Fig. 2). The food paradigm was performed towards the end of the imaging session (after approximately 35 min). This might have resulted in increased head motion (three subjects) and termination of the session by the participants (five). Spiking artifacts were also more likely to occur at the end of a scanning session, possibly due to the scanner heating up, requiring the exclusion of further 22 participants (Fig. 2). Seventy-six functional data sets could finally be analyzed: 31 AN, 18 REC and 27 NP.

### Post-hoc power calculation

A post-hoc power calculation for the food > non-food contrast with Random Field Theory control was performed separately for the final AN (power 0.81 with cluster-defining threshold *p* < 0.001, power 0.59 with cluster-defining threshold *p* < 0.01), REC (power 0.32 with cluster-defining threshold *p* < 0.001, power 0.24 with cluster-defining threshold *p* < 0.01) and NP (power 0.88 with cluster-defining threshold *p* < 0.001, power 0.54 with cluster-defining threshold *p* < 0.01) samples applying the “Neuro-Powertool” (http://neuropowertools.org/; retrieval date 05th May, 2022).

### Demographic and clinical characteristics

Patients with AN had typical features of psychopathology and a lower BMI compared to the REC and NP group. REC participants showed good restitution of all clinical data (Table 1). The NP group was matched to the AN group and therefore younger than REC (Table 1). Lowest BMI and duration of illness did not differ between REC and AN. Patients with AN consumed fewer calories than REC and NP, but did not differ in feeling of satiety.

**Table 1:** Demographic and clinical characteristics

	AN (n = 31)		REC (n = 18)		NP (n = 27)		ANOVA	Post hoc *t* test Tukey–Kramer*
	Mean	SD	Mean	SD	Mean	SD	*(F; p)*	
Age (years)	24.1	4.3	27.4	7.7	23.6	3.0	F = 3.7; *p* < 0.03	REC > NP
Current BMI (kg/m^2^)	16.3	1.4	20.8	1.2	22.1	2.2	F = 92.1; *p* < 0.001	NP > REC > AN
Lowest-lifetime BMI (kg/m^2^)	14.8	1.4	14.4	2.9	20.9	1.8	F = 40.85; *p* < 0.001	NP > REC,AN
Duration of illness (in months)	69.4	47.5	65.0	62.0	–	–	F = 0.076; *p* = 0.784	–
Duration of recovery (in months)	–	–	52.7	77.1	–	–	–	–
EDE—mean	3.3	1.0	0.7	0.4	0.4	0.3	F = 142.9; *p* < 0.001	AN > REC,NP
EDE—sum score	74.4	26.4	15.0	9.0	9.9	7.0	F = 111.1; *p* < 0.001	AN > REC,NP
EDI—total score	61.9	9.4	47.7	5.2	44.6	3.1	F = 51.85; *p* < 0.001	AN > REC,NP
EDI-2—*drive for thinness* (t-values)	83.6	20.0	48.0	13.3	44.6	6.4	F = 58.6; *p* < 0.001	AN > REC,NP
EDI-2—*body dissatisfaction* (t-values)	61.8	12.9	50.3	9.5	46.6	8.0	F = 15.8; *p* < 0.001	AN > REC,NP
STAI—*State-Score*	38.8	6.7	35.7	5.3	32.8	4.8	F = 7.9; *p* = 0.001	AN > NP
STAI—*Trait-Score*	45.6	7.7	31.9	7.9	29.3	6.8	F = 38.3; *p* < .001	AN > REC,NP
BDI-II	22.4	10.2	6.5	6.2	2.3	2.7	F = 59.2; *p* < 0.001	AN > REC,NP
MWT-B	28.4	5.4	29.8	4.9	28.0	4.3	F = 0.7; *p* = 0.477	–
Calorie intake at breakfast	160.1	180.7	300.6	124.9	386.3	85.7	F = 19.0; *p* < 0.001	NP, REC > AN
Satiety	6.4	1.8	6.6	1.7	6.7	1.0	F = 0.221; *p* = 0.802	–

*AN* anorexia nervosa, *BDI-II* Beck Depression Inventory II, *BMI* body mass index, *EDE* Eating Disorder Examination, *EDI-2* Eating Disorder Inventory-2, *NP* non-patients, *MWT-B* multiple-choice vocabulary intelligence test (German: Mehrfachwahl-Wortschatz-Intelligenztest), *REC* recovered AN, *SD* standard deviation, *STAI* State-Trait Anxiety Inventory

*p < 0.05 (cf [45])

### Within-group activation

**Fig. 3:**
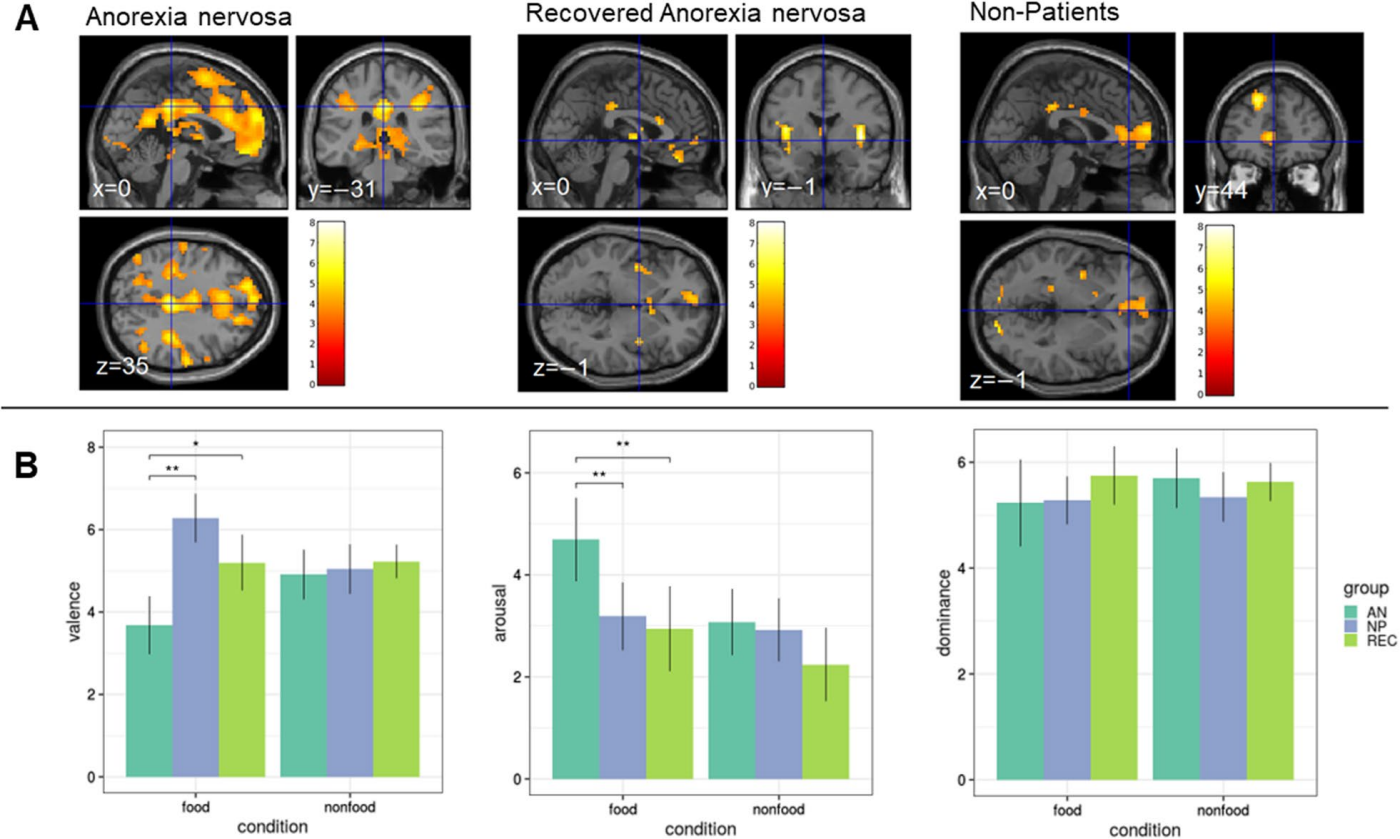
**A** T-maps of within-group differences for the contrast food > non-food for AN, REC and NP, *p*_uncorr._ < 0.001, k ≥ 10 voxels. Color bars represent the t-scores (white/yellow = high, red = low). **B** Behavioral data. Mean valence (0 = negative to 8 = positive valence), arousal (0 = unaroused to 8 = very aroused) and dominance (0 = not dominant to 8 = very dominant) ratings for the three groups (AN, REC, NP) and the two conditions (food and non-food). *p < 0.01, **p < 0.001. *AN* anorexia nervosa; *NP* non-patients, *REC* recovered AN (cf. [45])

The contrast food > non-food showed increased BOLD activation of frontoinsular cortices in all three groups (Table 2, Fig. 3a). Within-group analyses showed no significant results in any of the three groups for the contrast non-food > food.

**Table 2:** Within-group differences for the contrast food > non-food (cluster-defining threshold of *p*_uncorr._ < 0.001, k ≥ 10 voxels)

	Hemisphere	Voxels	*P*_FWE-corr._* Cluster*	*P*_FWE-corr._* peak voxel*	MNI			T-score
					*x*	*y*	*z*	
*Anorexia nervosa (AN)*								
Middle occipital gyrus	L	1086	< 0.001	0.001	− 21	− 97	8	7.05
Occipital gyrus	R			0.013	33	− 73	− 7	6.06
Lingual gyrus	R			0.015	15	− 88	− 4	5.98
	R			0.027	21	− 88	− 4	5.73
Inferior occipital gyrus	L			0.042	− 30	− 76	− 4	5.53
Insula	R	5474	< 0.001	0.004	0	− 31	35	6.63
Inferior occipital gyrus	L			0.008	− 12	20	65	6.27
Superior frontal gyrus	L			0.034	− 18	50	35	5.56
	R			0.029	15	38	50	5.70
	R			0.030	− 9	14	26	5.68
Posterior medial frontal cortex	L			0.028	− 6	53	38	5.71
	L			0.034	− 6	5	68	5.62
	R			0.044	9	17	68	5.50
Superior frontal gyrus, medial part	L			0.032	0	59	23	5.65
Middle frontal gyrus, orbital part	L			0.039	− 9	56	− 7	5.50
Medial cingulate cortex	L			0.045	− 9	− 7	32	5.49
Precuneus	L			0.046	− 6	− 52	20	5.44
Supramarginal gyrus	R	320	< 0.001	0.022	60	− 16	29	5.82
Precuneus	L	375	< 0.001	0.022	− 33	− 1	14	5.82
Postcentral gyrus	R	78	0.102	0.028^‡^	− 60	− 16	26	5.70
*Recovered anorexia nervosa (REC)*								
Insula	L	137	0.002	0.001	− 36	− 4	11	9.92
	L			0.007	− 39	5	− 13	8.38
	R	52	0.096	0.016^‡^	39	− 1	2	7.86
Superior orbital gyrus	L	299	< 0.001	0.008	− 21	35	− 13	8.32
Superior frontal gyrus	L	109	0.007	0.326^†^	39	− 1	2	5.67
Thalamus		104	0.008	0.387^†^	0	− 7	2	5.52
*Non-patients (NP)*								
Calcarine gyrus	R	114	0.014	0.005	18	− 94	5	6.91
Superior frontal gyrus	L	674	< 0.001	0.006	− 15	47	44	6.84
Superior occipital gyrus	L	244	< 0.001	0.012	− 18	− 91	2	6.51
Superior parietal lobule	L	196	0.001	0.012	− 24	− 76	47	6.50
Insula	L	197	0.001	0.013	− 36	− 7	11	6.47
Supramarginal gyrus	R	66	0.080	0.016^‡^	− 60	− 16	32	6.36

*FWE-corr.* family wise error corrected, *MNI* standardised brain according to Montreal Neurological Institute

^†^only significant at a cluster level, FWE-corrected

^‡^only significant at a peak/voxel level, FWE-corrected

### Between-group comparison

Applying the initial threshold of *p*_uncorr._ < 0.001, the two-sample t-test revealed no significant BOLD differences between groups for any of the comparisons (AN > REC, REC > AN, NP > REC, REC > NP).

At a lenient cluster-forming initial threshold of *p*_uncorr._ < 0.01, there were no differences between REC and NP neither. AN showed a higher BOLD response of an occipital area compared to REC (Additional file 2). Comparisons of AN and NP have been reported previously [45], where AN showed higher BOLD responses compared to NP in midcingulate, pre/postcentral and parietal areas when using a lenient initial threshold, and no significant group differences with recommended more stringent initial cluster-forming thresholds [45, 59]. Neither the SVC analysis for the insula nor for the amygdala ROIs survived family-wise error correction.

### Multiple regression analysis

None of the regression models yielded results that exceeded the significance threshold. Additional file 3 depicts the results of the performed multiple regression models for the affective ratings (arousal/valence).

### Behavioral data

The AN group had lower valence and higher arousal ratings compared to both REC and NP, while REC did not differ from NP (Table 3; Fig. 3b). There were no group differences in the ratings of non-food stimuli.

**Table 3:** Behavioral data

	AN		REC		NP		ANOVA F(df); p	post-hoc Tukey test *
	Mean	SD	Mean	SD	Mean	SD		
*Food images*								
Valence	3.70	1.85	5.20	1.36	6.28	1.43	17.98 (2); < 0.001	NP,REC > AN
Arousal	4.69	2.16	2.94	1.68	3.19	1.61	6.518(2); < 0.01	AN > NP,REC
Dominance	5.23	2.16	5.75	1.11	5.28	5.34	0.643 (2); 0.529	–
*Non-food images*								
Valence	4.91	1.59	5.23	0.82	5.04	1.46	0.289 (2); 0.75	–
Arousal	3.07	1.71	2.24	1.45	2.92	1.50	1.638 (2); 0.202	–
Dominance	5.70	1.49	5.62	0.73	5.34	1.13	–	–

0 = negative valence to 8 = positive valence, 0 = unaroused to 8 = very aroused, 0 = not dominant to 8 = very dominant. *AN* anorexia nervosa, *NP* non-patients, *REC* recovered AN

*p < 0.05 (cf. [45])

## Discussion

This cross-sectional fMRI study on functional neural correlates towards disease-related stimuli (food vs. non-food images) revealed no group differences between REC and NP with neither a conservative initial height threshold [45] nor a lenient one. REC and NP also showed no difference in affective ratings, i.e. with respect to the subjective experience of the stimuli. Furthermore, there was no significant correlation of the valence and arousal ratings with the BOLD response towards food stimuli in the insular cortex.

The lack of group differences between REC and NP in functional neural correlates to visual food cues is in line with results of previous studies, which also detected no differences between REC and NP [21–23]. However, some studies reported differences between groups [24–26], which was only the case in region-of-interest analyses, but not whole-brain in the Holsen et al. sample [25]. These inconsistencies are likely due to a variety of reasons, some of which are associated with limited reliability and replicability [45]. Further, it is likely that studies yielded false-positive results due to small sample sizes as well as different statistical methods (e.g., region of interest vs. whole brain analyses, statistical thresholds, etc.) [60, 61]. Comparability of studies is hampered due to *heterogeneity across participants* (BMI, duration of illness and recovery, etc.), *within participants* (daytime, hormonal level, etc.), and *across study sites* (study design, scanner hardware, etc.) [45, 62]. This, once again, supports the need of replication studies [63], especially in the field of fMRI [64, 65]. With respect to the early study of Uher et al. [24], which reported group differences of functional neural correlates and which used visual stimuli similar to ours, these factors likely account for the differing results.

Naturally, *recovery criteria* are another important topic with respect to state and trait. Recovery in AN in principle requires the absence of eating disorder psychopathology and a minimal BMI for a certain period of time [66–69], although the exact quantitative values vary.

For further detailed discussion of this topic with respect to recovery, remission and relapse see for example Khalsa et al. [69]. We used conservative measures in the current study, i.e. absence of eating disorder pathology for at least 12 months, an EDE score within one standard deviation of normal. Most REC were in the BMI ≥ 20 kg/m^2^ range with a few exceptions. In consequence, our sample of REC represents clinically stable long-term recovered individuals, which seems important in order to avoid cerebral aberrations and dysfunction—which might still be present after short term—due to insufficient clinical recovery.

However, most previous studies used such recovery criteria (see Additional file 1), and we could not detect any pattern with respect to the rather divergent study results.

We aimed to put behavioral and cerebral response to visual food cues in the context of state and trait. In contrast to AN, REC participants did not differ psychometrically or in their valence and arousal ratings to food stimuli from NP, indicating unimpaired cognitive and emotional processing of food stimuli. This is accompanied by a cerebral neural activation pattern which is similar to NP, i.e. which is not different between groups, even when applying a very lenient initial height threshold statistically. These data are comparable to two recent studies using a food paradigm [21, 23].

In contrast to previous studies [22, 25], correlation and interaction analysis showed no significant results. However, although our study has a larger sample size than the previous ones [22, 25], it is possible that the effects are still too small to be detected with the group size of this study [70].

Comparing REC and AN, experiential data differed. The BOLD responses showed no regions of higher activation of REC as suggested by Uher et al. [24]. Applying a lenient initial height threshold, we however also found increased activation in occipital areas in AN when compared to REC, but this was located on the left side, while in the other study it was right-sided [24]. With respect to the meaning of possible occipital differences, these do not represent brain areas associated with emotional and motivational processing, but visual processing and might be unspecific [43]. Finally, we point to a recent report on neurobiological markers of 55 REC, which did not find differences between NP and REC, which can be regarded as a complementary finding to our results [71].

Not only neuronal and glial damage appear to be state phenomena [4, 71] but, according to our results, neural processing and affective rating of food stimuli also seem to be state-related. Certain neuropsychological characteristics seem to persist in some patients with AN after clinical recovery, such as difficulties in set-shifting abilities and weaker central coherence [72]. Consequently, functional cerebral aberrations are likely no candidates for endophenotypes in AN.

The study has limitations. REC were slightly older than the other groups, which is due to the course of the illness. When including age as a covariate, the results remained significant. Larger cohorts and in particular longitudinal intra-individual designs will complement our knowledge — ideally through repeated measurements. However, AN is a disease with low prevalence and high chronicity, and therefore it is difficult to recruit large enough samples — in particular in single-center studies. A further limitation of the current study represents that AN consumed less calories before the imaging protocol — which, however, is also difficult to compensate/equalize methodically. Due to the differences in calorie intake between AN and REC/NP, food processing might be due to a temporary situation of hunger during measurement and not representing a stable finding in acute AN. Although our findings suggest a restitution of brain function with regard to food stimuli, the paradigm might not be sensitive enough to detect weaker effects. Furthermore, it cannot be ruled out that there may be other areas where abnormalities persist, such as in the perception of one's own body.

## Conclusions

In summary, similar to the restitution of structural cerebral abnormalities [5, 20] and serum neuronal biomarkers [71], functional brain aberrations also seem to be a *state phenomenon*, at least in terms of processing of food stimuli. However, this should be proven experimentally by longitudinal studies in AN and larger cohorts, which is not easily feasible methodically. Replicability is affected by several methodological issues, which we discussed in more detail elsewhere [45]. From a clinical perspective, the restitution of structural and functional cerebral alterations is an interesting issue concerning the transition of neuroscientific knowledge into clinical practice [73].

## Supplementary Information

**Additional** **file 1:** Overview of previous studies.

**Additional** **file 2:** Results of between-group differences for the contrast food > non-food.

**Additional** **file 3:** Results of the linear regression models for the affective ratings.

## Data Availability

Data are available from the senior authors on reasonable request.

## Abbreviations

AN: Anorexia nervosa
BDI: Beck depression inventory
BOLD: Blood oxygenation level dependent
BMI: Body mass index
EDE: Eating disorder examination
EDI: Eating disorder inventory
fMRI: Functional magnetic resonance imaging
FOV: Field of view
MPRAGE: Magnetization-prepared rapid gradient echo
MNI: Montreal Neurological Institute
MWT: Multiple choice vocabulary test
NP: Non-patients
REC: Recovered
SCID: Structured clinical interview for DSM
SPM: Statistical parametric mapping
STAI: State trait anxiety inventory
TE: Echo time
TR: Repetition time
