# Supplementary material for: State or trait: the neurobiology of anorexia nervosa — contributions of a functional magnetic resonance imaging study
Source: J Eat Disord. 2022 May 31;10:77. doi: 10.1186/s40337-022-00598-7 (PMC9158182; doi:10.1186/s40337-022-00598-7)
Supplement: Supplementary file 1 — Additional file 1: Overview of previous studies. [file 40337_2022_598_MOESM1_ESM.docx]

**Additional file 1: Overview of previous studies**

**I: Disorder-specific fMRI studies in patients with AN, REC and NP**

|  | **Authors** | **Sample** | **Age (years)** | **BMI (kg/m^2^)** | **Diagnostic criteria** | **Duration of illness (years)** | **REC criteria** | **Duration of REC (years)** | **Task** | **Results** | **Statistical methods** |
| --- | --- | --- | --- | --- | --- | --- | --- | --- | --- | --- | --- |
| ***food imgaes*** | ***Uher et al. 2003*** [24]***, Uher et al. 2004*** [43] | 8 AN  9 REC  9 NP | AN 25.6 (2.8)  REC 26.9 (5.3)  NP 26.6 (3.3) | AN 16.6 (1.2)  REC 20.4 (2.1)  NP 22.2 (3.8) | DSM IV | AN 12.5 (3.6)  REC 3.4 (1.3) | >85% of IBW, no changes >5% of body weight in the last two years, regular menstrual periods during the last year (at least 10 cycles); no psychotropic medication during the last year | 6.3 (3.2) | food and non-food images | ***WBA: food>non-food:*** AN>NP: l. ventromedial PFC, r. lingual cortex, AN<NP: l. inf. parietal lobule, l. cerebellum, AN<REC: r. apical, med. and lat. PFC, dACC, parieto-occipital cortex, l. cerebellum, AN>REC: r. occipital-lingual cortex, **REC>NP: r. and l. med. PFC, r. and l. ACC, l. cerebellum, REC<NP: l. inf. parietal lobule, l. visual occipital cortex** | WBA: voxel wise threshold: p<0.01, for group comparison cluster-wise difference p<0.001 to control for multiple comparisons |
|  | ***Holsen et al. 2012*** [25] | 12 AN  10 REC  11 NP | AN 21.8 (2.7)  REC 23.4 (2.3)  NP 21.6 (1.3) | AN 18.0 (0.8)  REC 22.1 (2.2)  NP 22.4 (1.3) | DSM IV | AN 5.0 (2.7)  REC 4.0 (2.3) | maitenance of 90 - 110 % of ideal body weight for at least 6 months | 3.5 (2.9) | food (high-calorie and low-calorie) and non-food images, premeal and postmeal | ***WBA: high-calorie vs. objects:*** AN<NP: *premeal:* r. ventral lat. ant. thalamic nucleus, l. centromedian thalamic nucleus, l. insular claustrum, l. PCC, l. precuneus, r. parietooccipital transitional zone, AN<REC: *premeal:* r. ventral lat. post. thalamic nucleus, *postmeal:* l. middle temporal gyrus, r. parietal operculum, l. paracentral lobule, **REC vs. NP: no significant results**  ***ROI: high-calorie vs. objects:*** AN<NP: *premeal:* l. hypothalamus, l. amygdala. l. hippocampus, r. OFC, r. and l. insula, *postmeal*: l. amygdala, l. insula, AN>REC: *postmeal:* r. amygdala, AN<REC: *postmeal:* r. and l. insula, **REC<NP:** *premeal:* **r. and l. hypothalamus, l. amygdala, r. insula** | WBA: cluster defining threshold: p_uncorr._<0.05, k≥10 voxels, p_FWE-corr._<0.05  ROI: voxelwise p_uncorr._<0.05 define significant clusters, ROIs signficant with p_FWE-corr._<0.1, voxelwise significance of p_FWE-corr._<0.05 |
|  | ***Sanders et al. 2015*** [26] | 15 AN  14 REC  15 NP | AN 25.6 (5)  REC 24.3 (5)  NP 25.8 (5) | AN 14.5 (1.7)  REC 21.1 (1.9)  NP 21.5 (2.3) | DSM IV | AN 7.8 (4.1)  REC 4.1 (2.7) | BMI: 18.5 - 25 kg/m^2^, no current dieting or weight loss, no behavior pattern of restrained eating in EDE-Q | 4.7 (2.7) | food (high-calorie and low-calorie) and non-food images | ***ROI:***  ***food>non-food:*** AN>NP: r. cerebellum, l. middle frontal gyrus, AN<NP: r. precuneus, r. sup. frontal gyrus; AN vs. REC: no signficant results**, REC>NP: r. caudate nucleus, r. cerebellum, l. middle frontal gyrus, l. postcentral gyrus** | ROI: fROI identified a posteriori with p_uncorr._<0,05, k>9 voxels of a priori identified ROIs, p_HSD-corr._<0.05 |
|  | ***Scaife et al. 2016*** [23] | 12 AN  14 REC  16 NP | AN 29.4 (6.0)  REC 27 (6.5)  NP 24.3 (5.7) | AN 15.4 (1.9)  REC 20.9 (1.6)  NP 21.2 (2.0) | DSM IV | AN 10.3 (5.2)  REC 5.8 (4.2) | BMI: 18.5 - 25 kg/m^2^, no eating disorder pathology in the last 12 months, EDE-Q scores within a standard deviation of global mean scores of young women | − * | food (high-calorie and low-calorie) images | ***WBA: food vs. baseline:*** AN<NP: r. and l. precentral gyrus, l. precuneus, r. sup. parietal lobule, ***high calorie:*** AN>NP: ant. PFC; ***low calorie:*** AN<NP: ant. PFC, dlPFC, supramarginal/lingual gyri; AN vs. REC: no significant results, **REC vs. NP: no significant results** | WBA:  p<0.05 including multiple-comparison corrections |
|  | ***Boehm et al. 2021*** [21] | 35 AN  33 REC  58 NP | AN 16.2 (3.5)  NP_AN_ 16.3 (3.3)  REC 22.2 (3.51)  NP_REC_ 21.8 (3.6) | AN 14.6 (1.5)  NP_AN_ 20.7 (2.2)  REC 20.64 (1.6)  NP_REC_ 21.3 (1.8) | DSM-5 | AN 1.1 (1.6) | BMI: >18.5 kg/m^2^ or > 10th age percentile for at least 6 months, menstruation, no binging, purging or restrictive eating patterns | 4.9 (4.7) | food and non-food images | ***GLM: food vs. non-food:*** AN>NP: inf. frontal junction, fusiform gyrus/parahippocampal gyrus, cuneus, **NP vs. REC: no significant results**  ***MVPA: food vs. non-food:*** classification accuracy within post. fusiform gyrus: AN>NP; **REC vs. NP: no significant results** | General linear model: group x stimulus condition x stimulus type interaction, cluster-level p_FWE-corr._<0.05  Multivoxel pattern analysis: cluster-defining threshold: p_uncorr._<0.001, p_FWE-corr._<0.05 |
| ***taste*** | ***Frank et al. 2016*** [31] | 21 AN  19 REC  20 BN†  19 OB†  27 NP | AN 22.9 (6.1)  REC 27.0 (5.3)  NP 26.2 (7.0) | AN 16.0 (1.1)  REC 20.2 (1.1)  NP 21.5 (1.4) | DSM IV | − * | normal weight, regular menses, normal exercise patterns for one year | − * | sucrose solution, no solution, artificial saliva | ***WBA: sucrose vs. artificial saliva:*** classification accuracy within insula: AN<NP, AN<REC**; REC vs. NP: no significant results** | WBA: p_FWE-corr._<0.05, k≥5 voxels |

ACC: anterior cingulate cortex, AN: Anorexia nervosa, ant.: anterior, BMI: Body-Mass-Index, BN: bulimia nervosa, corr.: corrected for multiple comparisons, dACC: dorsal anterior cingulate cortex, dlPFC: dorsolateral prefrontal cortex, EDE-Q: Eating Disorder Examination - Questionnaire, FWE-corr.: Family-Wise-Error-Correction, GLM: general linear model, HSD-corr.: honest-signficant-difference, IBW: Ideal Body Weight, inf.: inferior, lat.: lateral, l.: left, k: minimal cluster size, MVPA: multivoxel pattern analysis, med.: medial, NP: healthy controls, OB: participants with obesity, OFC: orbitofrontal cortex, PCC: posterior cingulate cortex, PFC: prefrontal cortex, post.: posterior, r.: right, REC: recovered AN, ROI: Region of Interest Analysis, uncorr.: uncorrected, WBA: Whole Brain Analysis, vs.: versus, *: not specified, †: results from experimental groups other than AN, REC or NP are not presented

**II: Disorder-specific fMRI studies with REC and NP**

|  | **Authors** | **Sample** | **Age (years)** | **BMI (kg/m^2^)** | **Diagnostic criteria** | **Duration of illness (years)** | **REC criteria** | **Duration of REC (years)** | **Task** | **Results** | **Statistical methods** |
| --- | --- | --- | --- | --- | --- | --- | --- | --- | --- | --- | --- |
| ***food imgaes*** | ***Oberndorfer et al. 2013*** [22] | 14 REC 12 NP | REC 28.9 (6.6)  NP 26.0 (6.8) | REC 22.0 (1.6)  NP 21.9 (1.0) | DSM IV | 7.4 (7.4) | >85 % of average body weight, regular menstruation, have not binged, purged or restricted food in the last year | 8.1 (4.8) | food and non-food images, conditioned stimulus (point or square) before unconditioned stimulus (image) | ***WBA: food:***  REC<NP: r. inf. cerebellum, l. sup. temporal gyrus; REC>NP**:** ant. and post. cerebellum, l. sup. frontal gyrus, l. sup. temporal gyrus, r. insula, l. PCC, r. inf. parietal lobule*;* ***food anticipation:***  REC<NP: l. inferior parietal lobule; REC>NP: med. and sup. frontal gyrus, dorsal striatum, putamen, thalamus, pulvinar, med. frontal gyrus; ***food>non-food anticipation:***  REC>NP: r. ventral ant. insula, ***food>non-food images:***  no significant results | WBA: food and non-food images and anticipation: p_uncorr._<0.05, food vs. non-food anticipation: p_uncorr._<0.01 for cluster≥1024 mm^3^, p_corr._<0.05 |
| ***taste*** | ***Wagner et al. 2008*** [30] | 16 REC 16 NP | REC 26.4 (6.2)  NP 26.8 (6.3) | REC 20.7 (2.2)  NP 22.9 (2.2) | − * | − * | >90 % of average body weight, regular menstrual cycles, have not binged, purged, restricted food in the last year | 3.8 (2.5) | sucrose solution and water | ***ROI:*** ***sucrose and water:*** REC<NP: insula, dorsal and middle caudate, dorsal and ventral putamen, ACC | ROI: defined a priori, p<0.05 |
|  | ***Cowdrey et al. 2011*** [27] | 15 REC 16 NP | REC 23.3 (3.5) NP 24.1 (2.9) | REC 21.3 (2.2) NP 21.2 (1.6) | DSM IV | − * | BMI of 18.5 - 25 kg/m^2^, regular menstruation, no use of psychoactive medication in the past 12 months, one standard deviation from the EDE-Q Score | 3.5 (2.4) | taste (chocalate, strawberry and tasteless) and image (chocalate, moldy strawberry and grey) | ***WBA:*** ***pleasant chocolate:***  REC>NP: *taste:* ventral striatum, putamen, PCC; *sight:* occipital cortex, ant. PFC, subgenual cingulate, med. PFC; *taste and sight:* pallidum; ***unpleasant strawberry:*** REC>NP: *taste:* insula, putamen; *taste and sight:* ACC, operculum, caudate, dlPFC | WBA: p_uncorr._<0.05, k≥30 voxel, p_FWE-corr._<0.05 |
|  | ***Oberndorfer et al. 2013*** [29] | 14 REC  14 RECb† 14 NP | REC 27.3 (1.4)  NP 27.4 (5.5) | REC 21.5 (2.8)  NP 22.6 (1.5) | DSM IV | REC 8.2 (1.7) | no restrictive eating or other pathological eating-related behaviors in the preceding 12 month, stable weight between 90% and 120% of ideal body weight for at least 12 months, regular menstrual cycles for the preceding 12 months | 5 (1.6) | sucrose and sucralose solution | ***ROI:***  ***sucrose:*** REC<NP: r. ant. insula ***sucralose:*** REC<NP: r. ant. insula | p_uncorr._<0.005, k≥32 voxels, p_corr._<0.05 |
|  | ***Radeloff et al. 2014*** [32] | 15 REC  14 RECb† 18 NP | REC 25.2 (4.0)  NP 24.7 (3.1) | REC 21.0 (2.4) NP 21.5 (1.8) | DSM IV | − * | normal BMI, regular menstrual cycles, no pathological behavior in eating for one year | 6.0 (2.6) | high fat stimulus, viscosity solution (CMC) and water | **WBA:** REC vs. NP: no signficant results  **ROI:** REC vs. NP: no significant results | WBA: p_uncorr._<0.001, p_FWE-corr._<0.05,  ROI: p_FWE-corr._<0.05 |
|  | ***Kaye et al. 2020*** [28] | 26 REC  22 NP | REC 26.2 (6.6) NP 25.7 (6.3) | REC 21.9 (1.7) NP 22.0 (2.1) | DSM IV | REC 5.9 (5.1) | no pathological eating behaviour or cognitions for one year, >85 % of average body weight, maintain weight stability, regular menstrual cycles | 5.6 (5.0) | „hungry“ condition and „fed“ condition, sucrose and water solution | **ROI:** group x condition analysis: l. ventral caudal putamen: NP: hungry>fed; REC: hungry<fed | ROI: voxelwise p_uncorr._<0.001, clusterwise p_corr._<0.05 (two-sided) |
| ***body*** | ***Kodama et al. 2018*** [13] | 12 REC  13 NP | REC 33.2 (0.0)  NP 29.7 (2.8) | REC 20.7 (0.7)  NP 21.5 (0.7) | DSM IV | − * | >85% of average body weight, regular menstrual cycles, no binge eating, purging, or significant restrictive eating patterns for at least one year | 5.6 (1.2) | body images: (1) comparison task (comparing own body with presented body) (2) weight estimation task of presented body | **WBA:** **(*1) comparison:*** REC>NP: l. sup. occicpital cortex; **(*2) estimation*:** REC<NP: r. middle temporal gyrus**;**  **ROI:** ***(1) comparison:*** REC>NP*:* l. pregenual ACC; **(*2) estimation:*** no significant results | WBA: height threshold p_uncorr._<0.001, extent threshold p_FDR-corr._<0.05,  ROI: defined a priori, small volume correction method, p_FWE-corr._<0.05 |

ACC: anterior cingulate cortex, ant.: anterior, BMI: Body-Mass-Index, corr.: corrected for multiple comparisons, dlPFC: dorsolateral prefrontal cortex, EDE-Q: Eating Disorder Examination - Questionnaire, FDR-corr.: False-Discovery-Rate-Correction, FWE-corr.: Family-Wise-Error-Correction, inf.: inferior, lat.: lateral, l.: left, k: minimal cluster size, med.: medial, NP: healthy controls, PCC: posterior cingulate cortex, PFC: prefrontal cortex, post.: posterior, r.: right, REC: recovered AN, RECb: recovered Bulimia nervosa, ROI: Region of Interest Analysis, WBA: Whole Brain Analysis, vs.: versus, *: not specified, †: results from experimental groups other than REC or NP are not presented

**III: Non-disorder-specific fMRI studies with AN, REC and NP**

|  | **Authors** | **Sample** | **Age (years)** | **BMI (kg/m^2^)** | **Diagnostic criteria** | **Duration of illness (years)** | **REC criteria** | **Duration of REC (years)** | **Task** | **Results** | **Statistical methods** |
| --- | --- | --- | --- | --- | --- | --- | --- | --- | --- | --- | --- |
| ***emotions*** | ***Uher et al. 2003*** [24]***, Uher et al. 2004*** [43] | 8 AN  9 REC  9 NP | AN 25.6 (2.8)  REC 26.9 (5.3)  NP 26.6 (3.3) | AN 16.6 (1.2)  REC 20.4 (2.1)  NP 22.2 (3.8) | DSM IV | AN 12.5 (3.6)  REC 3.4 (1.3) | >85% of IBW, no changes >5% of body weight in the last two years, regular menstrual periods during the last year (at least 10 cycles); no psychotropic medication during the last year | 6.3 (3.2) | emotionally aversive vs. neutral stimuli | ***WBA****:* no significant results | WBA: voxel wise threshold: p<0.01, for group comparison cluster-wise difference p<0.001 to control for multiple comparisons |
|  | ***McAdams et al. 2015*** [74] | 23 AN  10 REC  21 NP | AN 26.3 (8.1)  REC 29.6 (8.3)  NP 27.0 (6.1) | AN 18.0 (1.5)  REC 22.8 (2.7)  NP 22.8 (2.7) | DSM IV | − * | BMI>19 kg/m^2^ for >2 years, regular menstrual cycle, no hospital treatment for >2 years | − * | trust game | ***ROI:*** AN<NP: l. precuneus, r. angular gyrus, r. occipital gyrus, r. sup. frontal gyrus, l. fusiform gyrus; AN vs. REC: no significant differences; **REC<NP: l. precuneus, r. angular gyrus** | ROI: a posteriori identified ROIs with whole brain voxel-wise ANOVA (voxel wise p<0.005, k>105/111 voxel, cluster-level p_FWE-corr._<0.05) |
| ***self-perception*** | ***McAdams et al. 2016*** [75] | 22 AN  18 REC  19 NP | AN 27.6 (7.6)  REC 29.6 (8.1)  NP 27.9 (6.0) | AN 17.6 (1.5)  REC 22.8 (2.7)  NP 22.5 (2.4) | DSM IV | − * | BMI>19 kg/m^2^ for >2 years regular menstrual cycle, no hospital treatment for > 2 years | − * | social-identity-V2 task: (1) social self-identity (self-perception, self-relevance, evaluation), (2) faces task | ***ROI:* *(1) social identities:*** *self-identity*: MPFC-dACC: AN>NP, REC>NP, REC>AN; *self-relevance:* MPFC-Cing: NP>AN, NP>REC; *evaluation:* l. IFG/Insula: NP>AN, NP>REC, AN>REC; r. IFG/Insula, dACC: NP>REC, AN>REC; ***(2) faces task:*** l. fusiform/temporal gyrus: AN>NP, AN>REC, r. fusiform/temporal gyrus: AN>NP, AN>REC | ROI: a posteriori identified ROIs with whole brain voxel-wise ANOVA (voxel wise p<0.005, cluster-level p_FWE-corr._<0.05) |
| ***intimacy*** | ***Maier et al. 2019*** [35] | 31 AN  22 REC  35 NP | AN 24.1 (4.3)  REC 27.4 (7.1)  NP 22.8 (2.6) | AN 16.2 (1.4)  REC 20.6 (1.2)  NP 22.2 (2.3) | DSM 5 | AN 6.7 (3.6)  REC 7.8 (5.3) | BMI>20 kg/m², no eating disorder symptomatology for 12 months and EDE within one standard deviation of normal | 4.3 (5.6) | intimate vs. control images | ***WBA:*** AN>NP: r. dlPFC, AN<NP: l. and r. sup. parietal lobule; AN vs. REC: no signficant results; **REC vs. NP:** **no significant results** | WBA: cluster-defining treshold: p_uncorr._<0.001, k≥10, p_FWE-corr._<0.05 |
| ***reward learning*** | ***Olsavsky et al. 2019*** [36] | 28 AN  20 REC  20 BN† 43 NP | AN 22.9 (5.0)  REC 30.0 (8.0)  NP 26.4 (5.4) | AN 16.1 (1.0)  REC 20.7 (2.0)  NP 21.6 (1.5) | DSM IV | − * | normal weight, menstrual cycle, exercise and food intake for one year | − * | sucrose solution, no solution and artificial saliva as unconditioned stimulus, conditioned visual stimulus | ***WBA:*** AN>NP: l. ACC, r. supramarginal gyrus, r. middle temporal gyrus; AN>REC: l. ACC, r. supramarginal gyrus, r. middle temporal gyrus; NP vs. REC: no significant results  ***ROI:*** AN>NP: l. and r. ACC;  AN vs. REC: no significant results; **REC vs. NP: no significant results** | WBA: p_uncorr._<0.001, k≥100, p_FWE-corr._<0.05;  ROI: a priori defined ROIs, p<0.00625, p_FWE-corr._<0.05 |
| ***delay discounting*** | ***King et al. 2016, King et al. 2020*** [76,33] | 31 AN  31 NP  36 REC  36 NP | AN 15.7 (2.5)  NP 16.1 (2.4)  REC 22.2 (3.3)  NP 21.2 (3.4) | AN 14.7 (1.3)  NP 20.4 (2.0)   REC 21.1 (1.9)  NP 20.0 (1.6) | DSM IV | AN (current episode) 1.2 (1.9)  REC 2.9 (2.0) | BMI>18,5 kg/m, normal menstrual cycle, no binging, purging or restrictive eating in the last 6 months | 4.8 (3.4) | delay discouting | ***WBA:*** AN>NP: inf. occipital gyrus, thalamus; AN<NP: paracentral lobule, sup. frontal gyrus, inf. parietal lobule, PCC, precuneus, middle temporal lobule; **REC vs. NP: no significant results** | WBA: voxel-wise p<0.005, p_FWE-corr._<0.05 |
| ***fear*** | ***Maier et al. 2019*** [34] | 31 AN 23 REC  32 NP | AN 24.2 (4.3)  REC 27.1 (7.2) NP 23.7 (3.7) | AN 16.2 (1.4)  REC 20.6 (1.2)  NP 22.1 (2.5) | DSM IV | AN 6.9 (3.5) REC 7.2 (4.7) | BMI>20 kg/m², no eating disorder symptomatology for 12 months and EDE within one standard deviation of normal | 4.6 (6.1) | electrodermal stimulation as unconditioned stimulus, yellow and blue squares as conditioned stimulus | ***WBA***: AN<NP: ACC, r. IFG, r. insula; AN<REC: ACC, **REC vs. NP:** **no significant results** | WBA: cluster-defining treshold: p_uncorr._<0.001, k≥20 voxels, p_FWE-corr._<0.05 |

ACC: anterior cingulate cortex, AN: Anorexia nervosa, ant.: anterior, BMI: Body-Mass-Index, BN: Bulimia nervosa, corr.: corrected for multiple comparisons, dACC: dorsal anterior cingulate cortex, dlPFC: dorsolateral prefrontal cortex, EDE: Eating Disorder Examination, FWE: Family-Wise-Error-Correction, IBW: Ideal Body Weight, IFG: inferior frontal gyrus, inf.: inferior, lat.: lateral, l.: left, k: minimal cluster size, med.: medial, mPFC: medial prefrontal cortex, NP: healthy controls, PCC: posterior cingulate cortex, PFC: prefrontal cortex, post.: posterior, r.: right, REC: recovered AN, ROI: Region of Interest Analysis, WBA: Whole Brain Analysis, vs.: versus, *: not specified, †: results from experimental groups other than AN, REC or NP are not presented

**IV: FMRI studies with a longitudinal design**

|  | **Authors** | **Sample** | **Age (years)** | **BMI (kg/m^2^)** | **Diagnostic criteria** | **Duration and criteria of T2** | **Task** | **Results** | **Statistical methods** |
| --- | --- | --- | --- | --- | --- | --- | --- | --- | --- |
| ***working memory*** | ***Castro-Fornieles et al. 2010*** [40] | 14 AN_1_  14 NP_1_  9 AN_2_  14 NP_2_ | AN 15 (1.7)  NP 15.36 (0.1) | AN 14.9 (2.1)  NP − * | DSM IV | after 7 months of treatment and weight recovery (50. percentile), mean period 12.78 (12.10) months after T1 | working-memory-task | ***WBA:*** AN_1_>NP: l. sup. parietal lobule, l. inf. temporal gyrus; AN_1_>AN_2_: l. middle occipital cortex, l. angular gyrus, l inf. parietal lobule, l. frontal inf. operculum, r. and l. ACC, r. cerebellum; NP vs. AN_2_: no significant results | WBA: voxelwise p_uncorr._<0.001, k>10 voxel, p_FWE-corr._<0.05 |
| ***theory of mind*** | ***Schulte-Rüther et al. 2012*** [42] | 18 AN_1_ 22 NP_1_  18 AN_2_  16 NP_2_ | AN 15.7 (1.5) NP 15.8 (1.9) | AN_1_ 15.3 (1.5)  AN_2_ 18.1 (1.0)  NP_1_ 22.7 (3.9)  NP_2_ 22.8 (3.6) | DSM IV | at discharge from the hospital, mean period 107.1 (39.8) days after T1 | theory of mind task | ***WBA***: NP>AN_1_: r. sup. temporal gyrus; NP>AN_2_: l. middle temporal gyrus  **ROI:** NP>AN_1_: r. middle temporal gyrus, r. temporal pole; NP>AN_2_: r. temporal pole | WBA: voxelwise p_uncorr._<0.001, p_FWE-corr._<0.05;  ROI: p_FWE-corr._<0.05 |
| ***delay discounting*** | ***Decker et al. 2015*** [37] | 25 AN_1_ 21 NP_1_  18 AN_2_ 16 NP_2_ | AN 19.3 (2.5)  NP 20.7 (2.8) | AN_1_ 16.8 (1.4)  NP_1_ 21.4 (1.8) AN_2_ 20.2 (0.6)  NP_2_ 21.7 (1.8) | DSM 5 | after weight recovery (BMI>19.5 kg/m^2^) | delay discounting task | ***WBA:*** AN_1_ (vs. NP_1_) showed abnormal activation in dACC and striatum; AN_2_ (vs. NP_2_) showed increased activation in r. dlPFC, dACC, striatum, r. inf. parietal lobule  (NP_1_ and NP_2_ also differed significantly) | WBA: voxelwise p_uncorr._<0.01, p_corr._<0.01 |
|  | ***Doose et al. 2020*** [38] | 22 AN_1_  22 AN_2_ 22 NP | AN_1_ 15.5 (2.2)  AN_2_ 15.8 (2.2)  NP 15.5 (2.3) | AN_1_ 16.8 (1.4)  AN_2_ 20.2 (0.6)  NP_2_ 21.7 (1.8) | DSM-IV | after short-term weight restoration (>12% BMI increase) | delay discounting task | ***WBA:*** AN_1_>NP: inf. occipital gyrus; AN_1_<NP: lateral PFC, inferior parietal lobule, PCC/precuneus; AN_2_ vs. NP: no sigificant results | WBA: p_FWE-corr._<0.05 |
| ***reward learning*** | ***DeGuzman et al. 2017*** [41] | 21 AN_1_  21 NP_1_  21 AN_2_  21 NP_2_ | AN_1_ 16.4 (2.0)  NP_1_ 15.2 (2.4)  AN_2_ 16.5 (2.0)  NP_2_ 15.4 (2.3) | AN_1_ 16.4 (1.0)  NP_1_ 20.4 (2.4)  AN_2_ 18.7 (1.1)  NP_2_ 20.6 (2.4) | DSM-5  Mini-International Neuropsychiatric Interview | after treatment, mean period 42.29 (14.90) days after T1 | reward learning task | ***ROI: prediction error:*** AN_1_>NP_1_*:* r. and l. caudate, r. ventral and dorsal ant. insula, r. and l. post. insula; ***unexpected reward omission:*** AN_1_>NP_1_*:* l. caudate; ***unexpected reward receipt:*** AN_1_>NP_1_*:* r. dorsal ant. insula and post. insula; AN_2_>NP_2:_  r.and l. dorsal ant. insula, r. ventral ant. insula | ROI: a priori defined ROIs, Bonferroni correction, p_corr._<0.05 |
| ***set-shifting*** | ***Castro-Fornieles et al. 2019*** [39] | 30 AN_1_:  30 AN_2_ 16 NP | AN 14.9 (1.3)  NP 15.3 (1.4) | AN_1_ 16.9 (0.9) AN_2_ 18.9 (1.5)  NP 21.3 (2.1) | DSM 5 | after 6 - 7 months of treatment and after weight recovery | set shifting task | ***WBA:*** AN_1_<NP: l. middle and inf. occipital gyrus, cerebellum, l. fusiform gyrus, r. middle occipital gyrus, r. calcarine gyrus (results did not remain significant after FWE-correction); AN_2_ vs. NP: no sigificant results | WBA: p_uncorr._<0.001 , k≥10 voxel, p_FWE-corr._<0.05 |

ACC: anterior cingulate cortex, AN: Anorexia nervosa, ant.: anterior, BMI: Body-Mass-Index, corr.: corrected for multiple comparisons, dACC: dorsal anterior cingulate cortex, dlPFC: dorsolateral prefrontal cortex, FWE: Family-Wise-Error-Correction, inf.: inferior, lat.: lateral, l.: left, k: minimal cluster size, med.: medial, NP: healthy controls, PCC: posterior cingulate cortex, PFC: prefrontal cortex, post.: posterior, r.: right, REC: recovered AN, ROI: Region of Interest Analysis, T1: time of first measurement, T2: time of second measurement, WBA: Whole Brain Analysis, vs.: versus , *: not specified

**References**

13. Kodama N, Moriguchi Y, Takeda A, Maeda M, Ando T, Kikuchi H, et al. Neural correlates of body comparison and weight estimation in weight-recovered anorexia nervosa: a functional magnetic resonance imaging study. Biopsychosoc Med [Internet]. 2018 Oct 31 [cited 2020 Apr 25];12. Available from: https://www.ncbi.nlm.nih.gov/pmc/articles/PMC6208027/

21. Boehm I, Mohr H, King JA, Steding J, Geisler D, Wronski ML, et al. Aberrant neural representation of food stimuli in women with acute anorexia nervosa predicts treatment outcome and is improved in weight restored individuals. Transl Psychiatry. 2021 Oct 16;11(1):1–7.

22. Oberndorfer TA, Simmons A, McCurdy D, Strigo I, Matthews S, Yang T, et al. Greater anterior insula activation during anticipation of food images in women recovered from anorexia nervosa versus controls. Psychiatry Res [Internet]. 2013 Nov 30 [cited 2020 Jul 9];214(2). Available from: https://www.ncbi.nlm.nih.gov/pmc/articles/PMC3880160/

23. Scaife JC, Godier LR, Reinecke A, Harmer CJ, Park RJ. Differential activation of the frontal pole to high vs low calorie foods: The neural basis of food preference in Anorexia Nervosa? Psychiatry Res. 2016 Dec 30;258:44–53.

24. Uher R, Brammer MJ, Murphy T, Campbell IC, Ng VW, Williams SCR, et al. Recovery and chronicity in anorexia nervosa: brain activity associated with differential outcomes. Biological Psychiatry. 2003 Nov 1;54(9):934–42.

25. Holsen LM, Lawson EA, Blum J, Ko E, Makris N, Fazeli PK, et al. Food motivation circuitry hypoactivation related to hedonic and nonhedonic aspects of hunger and satiety in women with active anorexia nervosa and weight-restored women with anorexia nervosa. J Psychiatry Neurosci. 2012 Sep;37(5):322–32.

26. Sanders N, Smeets PAM, van Elburg AA, Danner UN, van Meer F, Hoek HW, et al. Altered Food-Cue Processing in Chronically Ill and Recovered Women with Anorexia Nervosa. Front Behav Neurosci [Internet]. 2015 Feb 27 [cited 2020 Apr 23];9. Available from: https://www.ncbi.nlm.nih.gov/pmc/articles/PMC4342866/

27. Cowdrey FA, Park RJ, Harmer CJ, McCabe C. Increased Neural Processing of Rewarding and Aversive Food Stimuli in Recovered Anorexia Nervosa. Biological Psychiatry. 2011 Oct 15;70(8):736–43.

28. Kaye WH, Wierenga CE, Bischoff-Grethe A, Berner LA, Ely AV, Bailer UF, et al. Neural Insensitivity to the Effects of Hunger in Women Remitted From Anorexia Nervosa. AJP. 2020 Jul 1;177(7):601–10.

29. Oberndorfer TA, Frank GKW, Simmons AN, Wagner A, McCurdy D, Fudge JL, et al. Altered insula response to sweet taste processing after recovery from anorexia and bulimia nervosa. Am J Psychiatry. 2013 Oct 1;170(10):1143–51.

30. Wagner A, Aizenstein H, Mazurkewicz L, Fudge J, Frank GK, Putnam K, et al. Altered Insula Response to Taste Stimuli in Individuals Recovered from Restricting-Type Anorexia Nervosa. Neuropsychopharmacol. 2008 Feb;33(3):513–23.

31. Frank GKW, Shott ME, Keffler C, Cornier MA. Extremes of Eating Are Associated With Reduced Neural Taste Discrimination. Int J Eat Disord. 2016 Jun;49(6):603–12.

32. Radeloff D, Willmann K, Otto L, Lindner M, Putnam K, Leeuwen SV, et al. High-fat taste challenge reveals altered striatal response in women recovered from bulimia nervosa: A pilot study. World J Biol Psychiatry. 2014 May;15(4):307–16.

33. King JA, Bernardoni F, Geisler D, Ritschel F, Doose A, Pauligk S, et al. Intact value-based decision-making during intertemporal choice in women with remitted anorexia nervosa? An fMRI study. J Psychiatry Neurosci. 2020 Mar 1;45(2):108–16.

34. Maier S, Schneider K, Stark C, Zeeck A, Tebartz van Elst L, Holovics L, et al. Fear Network Unresponsiveness in Women with Anorexia Nervosa. PPS. 2019;88(4):238–40.

35. Maier S, Spiegelberg J, Zutphen L van, Zeeck A, Elst LT van, Hartmann A, et al. Neurobiological signature of intimacy in anorexia nervosa. European Eating Disorders Review. 2019;27(3):315–22.

36. Olsavsky AK, Shott ME, DeGuzman MC, Frank GKW. Neural Correlates of Taste Reward Value Across Eating Disorders. Psychiatry Res Neuroimaging. 2019 Jun 30;288:76–84.

37. Decker JH, Figner B, Steinglass JE. On weight and waiting: delay discounting in anorexia nervosa pre- and post-treatment. Biol Psychiatry. 2015 Nov 1;78(9):606–14.

38. Doose A, King JA, Bernardoni F, Geisler D, Hellerhoff I, Weinert T, et al. Strengthened Default Mode Network Activation During Delay Discounting in Adolescents with Anorexia Nervosa After Partial Weight Restoration: A Longitudinal fMRI Study. Journal of Clinical Medicine. 2020 Apr;9(4):900.

39. Castro-Fornieles J, Serna E de la, Calvo A, Blázquez A, Moya J, Lázaro L, et al. Functional MRI with a set-shifting task in adolescent anorexia nervosa: A cross-sectional and follow-up study. Neuropsychologia. 2019 Aug 1;131:1–8.

40. Castro-Fornieles J, Caldú X, Andrés-Perpiñá S, Lázaro L, Bargalló N, Falcón C, et al. A cross-sectional and follow-up functional MRI study with a working memory task in adolescent anorexia nervosa. Neuropsychologia. 2010 Dec 1;48(14):4111–6.

41. DeGuzman M, Shott ME, Yang TT, Riederer J, Frank GKW. Association of Elevated Reward Prediction Error Response With Weight Gain in Adolescent Anorexia Nervosa. AJP. 2017 Feb 24;174(6):557–65.

42. Schulte-Rüther M, Mainz V, Fink GR, Herpertz-Dahlmann B, Konrad K. Theory of Mind and the Brain in Anorexia Nervosa: Relation to Treatment Outcome. Journal of the American Academy of Child & Adolescent Psychiatry. 2012 Aug 1;51(8):832-841.e11.

43. Uher R, Murphy T, Brammer MJ, Dalgleish T, Phillips ML, Ng VW, et al. Medial Prefrontal Cortex Activity Associated With Symptom Provocation in Eating Disorders. AJP. 2004 Jul 1;161(7):1238–46.

74. McAdams CJ, Lohrenz T, Montague PR. Neural Responses to Kindness and Malevolence Differ in Illness and Recovery in Women With Anorexia Nervosa. Hum Brain Mapp. 2015 Dec;36(12):5207–19.

75. McAdams CJ, Jeon-Slaughter H, Evans S, Lohrenz T, Montague PR, Krawczyk DC. Neural differences in self-perception during illness and after weight-recovery in anorexia nervosa. Soc Cogn Affect Neurosci. 2016 Nov;11(11):1823–31.

76. King JA, Geisler D, Bernardoni F, Ritschel F, Böhm I, Seidel M, et al. Altered Neural Efficiency of Decision Making During Temporal Reward Discounting in Anorexia Nervosa. Journal of the American Academy of Child & Adolescent Psychiatry. 2016 Nov 1;55(11):972–9.
