# Supplementary material for: State or trait: the neurobiology of anorexia nervosa — contributions of a functional magnetic resonance imaging study
Source: J Eat Disord. 2022 May 31;10:77. doi: 10.1186/s40337-022-00598-7 (PMC9158182; doi:10.1186/s40337-022-00598-7)
Supplement: Supplementary file 2 — Additional file 2: Results of between-group differences for the contrast food > non-food. [file 40337_2022_598_MOESM2_ESM.docx]

|  | Brain region | Hemis-phere | Voxels | P_FWE-corr._  *cluster* | P_FWE-corr._  *voxel* | MNI | | | T-Score |
| --- | --- | --- | --- | --- | --- | --- | --- | --- | --- |
|  |  |  |  |  |  | *x* | *y* | *z* |  |
| *AN > REC* | Fusiform gyrus | L | 561 | 0.011† | 0.533 | -33 | -58 | 11 | 3.99 |
|  | Calcarine gyrus | L |  |  | 0.609 | -21 | -58 | 8 | 3.90 |
|  | Middle occipital gyrus | L |  |  | 0.780 | -30 | -73 | 5 | 3.70 |
| *REC > AN* | No significant results | | | | | | | | |
| *NP > REC* | No significant results | | | | | | | | |
| *REC > NP* | No significant results | | | | | | | | |
| FWE-corr.: family-wise-error-corrected; † Cluster level, FWE-corrected. | | | | | | | | | |

**Additional file 2**

*Between-group differences for the contrast food>non-food, cluster-defining theshold of p_uncorr._<0.01, k≥10 voxels*
