# Supplementary material for: State or trait: the neurobiology of anorexia nervosa — contributions of a functional magnetic resonance imaging study
Source: J Eat Disord. 2022 May 31;10:77. doi: 10.1186/s40337-022-00598-7 (PMC9158182; doi:10.1186/s40337-022-00598-7)
Supplement: Supplementary file 3 — Additional file 3: Results of the linear regression models for the affective ratings. [file 40337_2022_598_MOESM3_ESM.docx]

**Additional file 3**

*Results of the linear regression models for the affective ratings (cluster-defining threshold of p_uncorr_.<0.001, k≥10 voxels)*

| dimension | contrast | Region | k_E_ | P_FWE-corr._  *cluster* | P_FWE-corr._  *peak voxel* | MNI | | | T-Score |
| --- | --- | --- | --- | --- | --- | --- | --- | --- | --- |
|  |  |  |  |  |  | *x* | *y* | *z* |  |
|  |  |  |  |  |  |  |  |  |  |
| *arousal* | Across all groups | Supplementary motor area right | 26 | 0.453 | 0.533 | 15 | 2 | 59 | 3.86 |
|  |  | Supplementary motor area left | 10 | 0.749 | 0.548 | -12 | -1 | 59 | 3.84 |
|  |  |  |  |  |  |  |  |  |  |
|  | REC>NP | Superior frontal right | 14 | 0.665 | 0.562 | 21 | 5 | 59 | 3.83 |
|  | NP>REC | - | - | - | - | - | - | - | - |
|  | REC>AN | Superior frontal right | 61 | 0.145 | 0.124 | 21 | 8 | 62 | 4.48 |
|  | AN>REC | - | - | - | - | - | - | - | - |
|  | NP>AN | - | - | - | - | - | - | - | - |
|  | AN>NP | - | - | - | - | - | - | - | - |
|  |  |  |  |  |  |  |  |  |  |
|  |  |  |  |  |  |  |  |  |  |
| *valence* | Across all groups | Supra marginal gyrus right | 37 | 0.311 | 0.435 | 60 | -22 | 17 | 3.98 |
|  |  | Inferior frontal gyrus pars triangularis right | 15 | 0.646 | 0.533 | 51 | 35 | 5 | 3.87 |
|  |  | Precentral right | 33 | 0.356 | 0.669 | 54 | 2 | 29 | 3.72 |
|  |  |  |  |  |  |  |  |  |  |
|  | REC>NP | Inferior frontal pars triangularis left | 10 | 0.752 | 0.742 | -39 | 14 | 23 | 3.64 |
|  | NP>REC | - | - | - | - | - | - | - | - |
|  | REC>AN | - | - | - | - | - | - | - | - |
|  | AN>REC | - | - | - | - | - | - | - | - |
|  | NP>AN | - | - | - | - | - | - | - | - |
|  | AN>NP | Insula right | 27 | 0.436 | 0.309 | 30 | 29 | 5 | 4.14 |
|  |  | Putamen right | 10 | 0.752 | 0.582 | 36 | -1 | 2 | 3.82 |
|  |  | Inferior frontal pars opercularis right | 13 | 0.687 | 0.789 | 39 | 14 | 29 | 3.58 |

FWE-corr.: family wise error corrected, k_E:_ cluster size, MNI: standardised brain according to Montreal Neurological Institute
